# Emergencies: on the misuse of government powers

**DOI:** 10.1007/s11127-021-00918-6

**Published:** 2021-07-22

**Authors:** Christian Bjørnskov, Stefan Voigt

**Affiliations:** 1 Department of Economics, Aarhus University, Fuglesangs Allé 4, 8210 Aarhus V, Denmark; 2 Research Institute of Industrial Economics (IFN), P.O. Box 55665, 102 15 Stockholm, Sweden; 3 Institute of Law and Economics, University of Hamburg, Johnsallee 35, 20148 Hamburg, Germany; 4 CESifo, Munich, Germany

**Keywords:** Constitutional emergency provisions, State of emergency, État de siege, Regime transformation, Positive constitutional economics, K40, Z13

## Abstract

Nine out of 10 constitutions contain explicit emergency provisions, intended to help governments cope with extraordinary events that endanger many people or the existence of the state. We ask two questions: (1) does the constitutionalization of emergency provisions help governments to cope with disasters and other extraordinary events? (2) What particular parts of emergency constitutions fare best? We find that the more advantages emergency constitutions confer to the executive, the higher the number of people killed as a consequence of a natural disaster, controlling for its severity. As this is an unexpected result, we discuss a number of potential explanations, the most plausible being that governments use natural disasters as a pretext to enhance their power. Furthermore, the easier it is to call a state of emergency, the larger the negative effects on basic human rights. Interestingly, presidential democracies are better able to cope with natural disasters than parliamentary ones in terms of lives saved, whereas autocracies do significantly worse in the sense that empowerment rights seriously suffer in the aftermath of a disaster.

## Introduction

Today, nine out of 10 national constitutions contain explicit emergency provisions. Declaring a state of emergency usually has two implications: (1) a shift in the balance of powers away from both the legislative and the judicial branches of government toward the executive branch; and (2) a reduction in citizens’ civil and political rights. Between 1985 and 2016, at least 137 countries declared a state of emergency (SOE) at least once. During the first wave of the COVID-19 pandemic in the spring of 2020 alone, 99 governments declared SOEs (Bjørnskov & Voigt, 2021). Although emergency provisions are common, it is amazing how little we know about the effectiveness of emergency constitutions.

This paper is a first attempt to analyze their effectiveness. For simplicity, we refer to all constitutional provisions dealing with any type of emergency as a country’s ‘emergency constitution’. Our analysis is confined to the effectiveness of emergency constitutions enacted to deal with natural disasters such as earthquakes, floods, droughts or pandemics. We do so because natural disasters truly are exogenous events, yet their direct consequences and side effects depend on governmental policies and reactions. We ask whether differences found in various emergency constitutions have discernible effects on the number of people killed as a direct consequence of a natural disaster. Because individual rights are likely to suffer under an SOE, we also ask whether the maintenance of individual rights, policies and democracy differ based on the specific wordings of emergency constitutions.

We are here concerned with the functionality of emergency constitutions: do certain aspects of emergency constitutions work as officially intended, in the sense that fewer people are killed in disasters? Or is a tradeoff evident in the sense that some constitutional provisions are associated with efficient disaster relief but simultaneously with a deterioration in the extent to which individual rights are being protected?

The worst SOE scenario is that the chief executive is not only unable to minimize the loss of life in the wake of a natural disaster, but effectively curtails individual rights. Although such a possibility might appear unlikely, numerous examples attest to such an outcome being a real possibility. For example, when a hurricane battered the Dominican Republic in 1931, Rafael Trujillo not only declared martial law and imposed “emergency taxes” on all citizens, but he also exploited the natural disaster to turn his regime into one of the longest-lasting dictatorships in the history of Central America (Crassweller, 1966). Trujillo is far from unique; any number of Latin American presidents used emergency provisions in comparable ways. For example, Wright (2014) counts 292 decrees issued under SOEs by the presidents of Bolivia, Ecuador, and Peru between 2000 and 2010.

The behavior of governments during the COVID-19 pandemic offers a plethora of examples of misused emergency provisions. Censoring freedom of expression is probably the most frequently reported misuse of power; beyond that several countries prorogued their legislative assemblies (e.g., Mauritius, Thailand and Serbia). Other countries suspended court operations (e.g., Bangladesh and Nigeria). When legislatures, judiciaries, or both, are prevented from monitoring the executive branch, the separation and balance of powers is threatened. In a few countries, military forces were marshalled to enforce governmental decrees (e.g., Iran, Malaysia and Denmark).^1^

The perspective of political economy may help us make sense of such behavior, as disasters might allow self-serving governments to expand their powers.^2^ Rather than exercising the additional powers granted to them under an SOE to save lives, the powers are taken advantage of to censor criticism.

In one of the very few studies that examine the effects of emergency constitutions, Davenport (1996) asks if political repression exercised by a government can be predicted by the explicit mentioning of either martial law or SOE in a country’s constitution. He finds that the existence of what we term an ‘emergency constitution’ significantly lowers a government’s willingness to resort to political repression, thereby constraining governments even when they are under domestic pressure. Keith and Poe (2004) build on Davenport’s (1996) analysis and focus on how domestic turmoil impacts respect for physical integrity, i.e. basic human﻿, rights. Contrary to Davenport (1996), they find that emergency provisions imposing tighter constraints on the executive branch are associated with more human rights’ abuses. Despite those contradictory findings, Bjørnskov and Voigt (2020) report evidence that the specific details of an emergency constitution do influence governmental reactions to threats.

We extend the scant literature in several ways. Davenport (1996) is interested only in whether an emergency constitution exists; we also are interested in whether it is implemented in actual practice. While his focus is on political repression, we consider other variables, such as the rule of law and democracy. Our analysis covers the post-Cold War era, beginning in 1990 and ending in 2011; our results are based on up to 1511 observations covering up to 122 countries.

Because we focus on SOEs instigated by natural disasters, and because one purpose of an emergency constitution is to empower the chief executive to save human lives, we explore whether the varying details of emergency constitutions are correlated systematically with the number of people who are killed by a natural disaster. We control for the severity of the disaster by taking the number of people affected explicitly into account. We find that the more benefits an executive enjoys after having declared an SOE, the *larger* is the number of people killed. Furthermore, we find that basic human rights are likely to suffer more the easier it is for governments to declare an SOE, and that the effect is more pronounced the more serious is the disaster. Finally, autocracies (of any type) are significantly more likely to curtail empowerment rights (i.e., freedoms of movement, speech, religion and political participation) than parliamentary democracies. An interesting correlation exists between our empirical findings and the results of a recent study that examines the relationship between violations of democratic principles during the COVID-19 pandemic and a country’s public health performance. Maerz et al. (2020, p. 11) find that “pandemic-driven violations of democratic standards in the name of human life are unjustified and lack empirical merit.”

Section 2 lays a foundation for the issue at hand and proposes two hypotheses dealing with the possible effects of declaring an SOE, one focusing on constraining the direct effects of natural disasters, the other dealing with potential human rights’ repercussions. Our data and descriptive statistics are presented in Sect. 3; our estimation approach is discussed in Sect. 4. The actual estimates are reported in Sect. 5. Section 6 concludes.

## Hypotheses

We propose to think of emergency constitutions as having two overarching goals: to minimize the negative effects of events that caused the declaration of an emergency and to reestablish the *status quo ante* in the medium to long run. When analyzing the effectiveness of emergency constitutions, both goals should, hence, be considered. From among the possible long-term goals, we focus explicitly on the protection of basic human rights here because they often seem to suffer in the aftermath of a state of emergency.

It is conceivable that some short-term effects need to be traded off against long-term effects. For example, a justification for the temporary derogation from civil and political rights might be that doing so will enable government to deal more effectively with the emergency. If a tradeoff exists, such short-term derogations should lessen the time needed to return to pre-event levels and reduce casualty rates during natural disasters (cf. Ignatieff, 2013). Regardless of the normative considerations, which are beyond the scope of our first take on the effects of emergency constitutions, any such constitution is based implicitly on two conjectures. The writers of constitutions posit that constitutionalizing emergency provisions is better than not constitutionalizing them.^3^ The fact that most national constitutions contain emergency provisions (Elkins et al., 2009) gives that conjecture some traction. The second implicit conjecture is that a temporary concentration of powers in the executive branch along with limits on some individual rights, increases the probability of the state’s survival. The fact that most of the countries without constitutionalized emergency provisions have passed statutory laws dealing with emergency provisions supports that conjecture. Before generating hypotheses on the possible effectiveness of different types of emergency constitutions, we want to be as precise as possible about the problem, which we structure on the relationship between three factors: events, institutions and actors.

### Events

To justify the declaration of an SOE, an event must be significantly relevant for the people affected directly as well as for the broader society. Although any number of events are possible, we focus on natural disasters because they are the most exogenous type. That does not imply that the consequences of a natural disaster are determined exogenously. For example, the thoroughness and enforcement of a country’s building codes likely will impact both the number of people affected and the death toll of an earthquake (Escaleras et al., 2007).

Although declaring an SOE in response to an exogenous natural disaster may be less contentious than declarations following other event types,^4^ other consequences are possible. If it is easier to declare an SOE in response to a natural disaster because doing so is innocuous, then governments that desire the discretionary executive powers offered under an SOE may take advantage of disasters as pretexts to gain such powers. On the other hand, the repercussions (e.g., basic human rights abuse) of declaring an SOE in response to endogenous event types may be more evident because a declaration gives the government more preemptive control.

We follow a conventional delineation and distinguish between four types of natural disasters: biological, geophysical, hydrological and climatological.^5^ Even after distinguishing between types of disasters, other variables must be considered, such as areas most likely to be affected, the ability to anticipate and adjust to a disaster, the speed at which a disaster develops, and the ratio of people killed to people affected by the disaster.

### Institutions

We are primarily interested in the effectiveness of emergency constitutions, i.e., the extent to which *de jure* institutions enable governments to achieve the goals mentioned above. The basic notion behind emergency constitutions is that extraordinary events require extraordinary measures not at the disposal of the executive branch during normal times. Since most emergency constitutions contain at least one of four extraordinary measures intended to mitigate the effects of natural disasters, we assume that the constitutional assemblies believe that those measures will allow the government to respond more effectively to a natural disaster.

1. Swift action usually is of the essence, which is why some constitutions enable the chief executive to dissolve parliament.
2. It has been argued that certain basic rights make swift government action more difficult, which is why many emergency constitutions enable the government to suspend them under an SOE.^6^
3. It has been argued that to save lives during and after a natural disaster, governments should have the right to infringe private property rights. Such emergency powers include trespassing on privately owned land and expropriating privately owned assets such as boats or trucks.
4. Some emergency constitutions allow government censorship of the media, based on the assumption that uncensored media reporting could impede fast and effective relief measures.

Those four measures can be thought of as the benefits an executive enjoys after an SOE is declared. But before the benefits can be enjoyed, an SOE needs to be declared and emergency constitutions contain rules that must be complied with constitutionally for declaring an SOE. Emergency constitutions can, first, make declaring a state of emergency easy or harder. When an SOE that can be declared by one actor only but requires the approval of others (such as the legislature, possibly even both houses), the declaration is “costly” and we expect fewer declarations. If the high costs are accepted and agreement is widespread in favor of an SOE, we also expect the chances of effective government response to be high. But the consent of veto players may not be the only requirement a government must meet, as many constitutions enumerate the events that justify declaring an SOE. In the empirical section, we analyze such provisions both singly as well as in the aggregate.

### Actors

The outcomes we explore are determined not only by the specific event type and emergency constitution, but also by two concrete policy choices. First, the chief executive needs to decide whether to declare an SOE.^7^ Secondly, the executive must respond to the emergency irrespective of declaring an SOE or not.

The effects that we are interested in (from the number of people killed to the effects on basic human rights) are determined by the interplay of three factors: the type and size of the natural disaster, the content of extant emergency constitutional provisions, and the government’s policy choices based on the emergency provisions, including the decision of whether or not to declare an SOE.

**Fig. 1:**
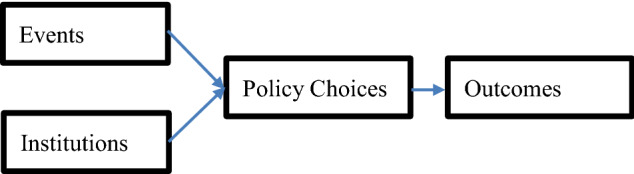
The interplay between events, institutions, and policy choices

Figure 1 illustrates the interplay between events, institutions, policy choices, and outcomes such as damaged infrastructure, lost lives and economic damage. Obviously, the type and scope of a natural disaster will affect our two outcomes of interest. Both outcomes also will be influenced by governmental behavior, i.e., policy choices, which, in turn, are bounded by the institutional constraints embedded in emergency provisions and democratic veto institutions, be they constitutionalized or not.

Although we focus only on natural disasters, numerous strategies are possible in choosing an appropriate estimation approach. We first offer two hypotheses on the effects of emergency constitutions. We begin with the obvious hypothesis that emergency constitutions work as intended:

#### Hypothesis 1

The more benefits an emergency constitution confers on the chief executive, the more effectively that branch will be able to deal with a disaster, implying fewer fatalities.

After an earthquake or a flood happens, it is essential that rescue operations start as quickly as possible. If government can ignore private property rights temporarily (trespassing on privately held land, forcing owners to support rescue operations with privately owned boats, and expropriating or coopting other assets) the effectiveness of its operations might increase. The same might be true if government has the competence to order medical personnel to support the rescue efforts, or to reallocate substantial budgetary items to the relief efforts without having to secure legislative consent, as the US emergency constitution allows. But we are interested not only in the effectiveness of rescue operations but also in their possible side effects, and particularly in the effects of government behavior on the extent to which basic human rights suffer.

#### Hypothesis 2

The easier it is for the executive branch to suspend basic human rights under an SOE, the more basic human rights are likely to suffer once an SOE has been declared.

The downside of granting government additional competences under an SOE might be that basic human rights suffer *without* facilitating rescue efforts. Democratic and autocratic governments alike may prefer to wield executive powers as widely as possible and thus have a vested interest in calling an SOE to exercise its emergency powers. A disaster thus may be a serendipitous event for such executives by opening a constitutional door to additional powers that they can misuse for other purposes.

The foregoing hypotheses rest on the assumption that emergency provisions are respected de facto and applied as intended. Hence, if a government is united in trying to limit the negative consequences of natural disasters, we do not expect the costliness of declaring an SOE to affect responses to natural disasters negatively. As spelled out above, political reactions to natural disasters normally are uncontroversial and reaching consensus across the political spectrum therefore can be assumed.^8^ The benefit aspects, on the other hand, may be associated with weaker respect for human rights during emergencies. Yet, to the extent that the provisions are used merely as pretexts for political action, we expect that stronger emergency provisions could even be associated with worse disaster consequences.

## Data and descriptive statistics

To test the effectiveness and potential side effects of emergency constitutions following natural disasters, we draw data from a diverse set of sources.

### Dependent variables of interest

For information on natural disasters and their severity, we rely on the Emergency Events Database (EM-DAT) (Guha-Sapir et al., 2014). We limit our identification of disasters by selecting only those events that affect at least one in every thousand citizens in the country (on average 1000 people).^9^ The total number of affected people determines event size; country-level observations are considered only for years in which EM-DAT records at least one natural disaster. Our empirical strategy avoids censoring problems and ensures that our results are not influenced by ‘fake’ SOEs declared without any natural disaster actually occurring.

Our main dependent variable is the (log) number of people killed per year by natural disasters. Our descriptive statistics are presented in Table 1, which shows that the means across disaster types are remarkably similar, with biological disasters the only slight “outlier”.

**Table 1:** Descriptive statistics

Variable	Mean	SD	Observations
Log killed, all disasters	3.120	2.381	2582
Log killed, Biological	3.555	4.589	2582
Log killed, Geophysical	2.991	2.416	2582
Log killed, Hydrological	3.129	2.383	2582
Log killed, Climatic	3.575	2.414	2582
Log affected, all disasters	9.067	4.589	2582
Log affected, Biological	8.792	4.732	2582
Log affected, Geophysical	8.693	4.847	2582
Log affected, Hydrological	9.105	4.569	2582
Log affected, Climatic	8.233	5.187	2582
Emergency constitution	0.733	0.442	2559
Physical Integrity rights	4.514	2.286	2395
Empowerment rights	8.523	3.923	2394
SOE declared	0.229	0.453	2507
Log population	9.348	1.717	2440
Log area	12.210	2.085	2575
Log latitude	2.928	.896	2561
Log GDP per capita	8.276	1.318	2376
Log openness	4.165	0.604	2395
Log gov. expenditures	2.2598	0.557	2395
Relative investment price	1.104	0.570	2395
Federal	0.126	0.332	2562
Mixed democracy	0.128	0.334	2480
Presidential democracy	0.223	0.416	2480
Civilian autocracy	0.269	0.444	2480
Military dictatorship	0.127	0.334	2480
Royal dictatorship	0.029	0.167	2480
Cost INEP	0.431	0.195	1593
Benefit INEP	0.224	0.183	1593
Democratic, 1950–60	3.265	4.689	2582
Predom. Protestant	0.072	0.258	2424
Predom. Muslim	0.218	0.413	2424
Common law	0.219	0.414	2562
Civil law	0.751	0.433	2562
Log coastline (km)	5331.861	19,961.830	2582
Low elevation (meters)	42.128	176.431	2582
High elevation	3319.296	2064.619	2582
Landlocked	0.229	0.419	2582

To assess the effects of emergency constitutions on human rights scores, we rely on two indicators developed by Cingranelli and Richards (2004). Their two indicators, a Physical Integrity Index and an Empowerment Index, aggregate 16 categories measuring basic human, political and civil rights.^10^ Furthermore, we explore the consequences of emergency constitutions on disaster-relief policies by relying on four variables from the Heritage Foundation’s (2016) Index of Economic Freedom: government size, rule of law, market openness and regulation. We also control for the extent of democracy, for which we employ Vreeland’s (2008) correction of the Polity IV Index, denoted ‘xpolity’.

### Explanatory variables

To determine effectiveness of emergency constitutions in handling natural disasters, we rely on the six dimensions included in the Index of Emergency Powers (INEP), first introduced in Bjørnskov and Voigt (2018a). The INEP contains of six dimensions. Three of them represent the cost side of declaring an SOE and three of them capture the benefits government enjoys after having declared an SOE. From a legal perspective, the first three cover procedural aspects, and the latter three deal with substantive provisions.

The cost dimensions account for (1) who has the power to declare a state of emergency, (2) who needs to consent to a declaration, and (3) what grounds legitimize a declaration. The benefit aspects ask whether the chief executive (1) has the power to dissolve parliament, (2) legally can compromise various human rights, and (3) whether private property rights as well as media freedom can be curtailed.

The six variables are coded based on a country’s constitution and each can take a value between 0 and 1, where 1 implies more benefits (or lower costs) for the chief executive. We rely on those variables to create two indicators. To code the overall indicator, we sum the values of all six variables and divide it by six, such that the overall INEP has a value between 0 and 1. In the empirical analysis, we calculate separate cost and benefit indicators using the INEP. That is done with the three relevant variables and calculated in the same way as the overall INEP. We enter the different indicators to determine whether the relative ease of declaring an SOE (cost) or the relative allocation of powers to the executive branch (benefit) determine how effectively the chief executive deals with a natural disaster.^11^ In addition, we enter a dummy variable to capture whether an SOE has been declared in a specific year. The INEP is based on information on constitutions in the Comparative Constitutions Project (Elkins et al., 2009), and our measure concerning the declaration an SOE is based on an update of the database in Hafner-Burton et al. (2011).

### Covariates

Following Kahn’s (2005) approach, we gather covariates on economic, geographic, and political factors. Our economic covariates include real income per capita in log form, trade openness, investment cost (the price of capital goods relative to the general price level), and government expenditures (relative to GDP) as a measure of general government activity. Our assumption is that richer countries are better equipped to deal with disasters. Openness might affect the diversity of a country’s economy; it likewise captures established trade routes that facilitate aid flows following a disaster. We also enter investment prices, defined above, as a measure of an economy’s structure. Specifically, if capital equipment is important in dealing with natural disasters and their immediate consequences, countries with relatively high relative capital prices may be likely to cope better by having more state and private capacity. All covariates are based on Heston et al. (2012).

Our political covariate is form of government. We assume that democratic and autocratic governments will react differently to disasters and that the latter will abuse emergency constitutions more frequently. If those suppositions are correct, autocratic countries dealing with a natural disaster will experience more deaths and more severe infringements on human rights. To make precise inferences, we distinguish democracies as parliamentary, mixed and presidential, and autocracies as civilian, military and royal. Our source for those fine-grained delineations is Cheibub et al. (2010), updated by Bjørnskov and Rode (2020).^12^

Our geographic covariates include population in log form, the log area of the country, and the log of the country’s latitude. Latitude is thought to be a good proxy for state capacity. The population data are from Heston et al. (2012); area and latitude data are from CIA (2015).

Our panel dataset and the period of our analysis (1990–2011) yield a maximum of 1511 observations from 122 countries; an SOE was declared in 23% of all country-year pairs. The period is capped by the end of the Cold War in 1990, along with the availability of data on SOE declarations and human rights by 2011. In all cases, we estimate by OLS with random effects and a lagged dependent variable to determine the effects of characteristics of emergency constitutions in natural disasters, which we include to make sure that fatalities are not wrongly attributed when, for example, disasters either last more than one year or extend beyond January 1. Simultaneously, this lagged dependent also serves as a control for a country’s proneness to disasters, such that we cannot interpret the estimate directly because it conflates two different factors.

## Our estimation approach

In order to obtain a clean estimate of the effects of emergency constitutions on disaster outcomes, we enter year fixed effects into our specification to control for joint global developments, dummies for legal origins (civil and common law) that might have an impact above and beyond the variables explicitly taken into account, and region fixed effects (Asia, Latin America and the Caribbean, the Middle East and North Africa, the Pacific Rim, and the post-communist countries) to limit the possibility that our estimates are tainted by region-specific differences. As such, we estimate Eq. (1), wherein *INEP* refers to our two indicators, *X* is a vector of all control variables and *D* is a matrix of fixed effects. When exploring side effects, we enter one of the measures of human rights, democracy or economic freedom as a left-hand side variable instead of the logarithm of the number of people killed.

1 $$\ln killed = \upalpha +\upbeta \ln affected +\upgamma INEP +\updelta X +\upupsilon D +\upvarepsilon$$

Our estimation strategy rests on the assumption that two countries treated with an identical event could have declared an SOE or not. The question is whether a particular type of emergency constitution clearly outperforms the other based on the goals introduced above. Relying on the characteristics captured by the INEP allows us to answer the question whether a particular constellation of competences outperforms others that also are realized empirically. In the next section, we estimate their effects using OLS with annual fixed effects. Because our dependent variable is entered in logarithmic form, the coefficient estimates pertaining to all other logged variables can be interpreted as elasticities, while the log of the affected population normalizes all effects relative to the size of the disaster.

While causality can never be established perfectly, we enter a lagged dependent variable to rule out the possibility that constitutional characteristics simply reflect the immediate responses to the threat environment. In addition, including the lagged dependent ensures that our estimates do not simply reflect past repressions of human rights creating more deaths during natural disasters.^13^ However, the entering lagged dependent variables on the right-hand side does not fully alleviate our concern of simultaneity bias, which would arise if the design of emergency constitutions *reflects* the natural threat environment. In other words, if the constitution’s drafters took explicitly into account the risks and/or the types of disasters naturally occurring in their nations, and did so in effective ways, neither the chances of observing an event nor the number of affected individuals would be exogenous.^14^

**Fig. 2:**
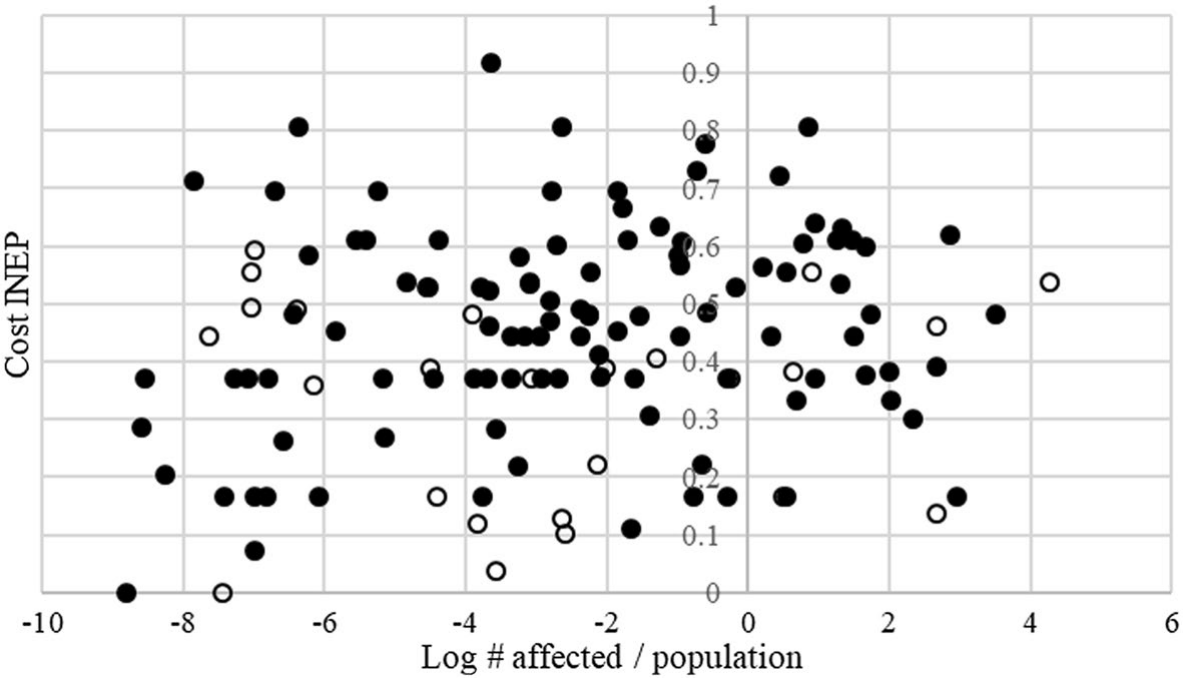
Average number of affected individuals, share of total population, versus Cost INEP. *Note*: Solid circles are democracies, and open circles are autocracies. The cost INEP captures the ease with which an SOE can be declated

**Fig. 3:**
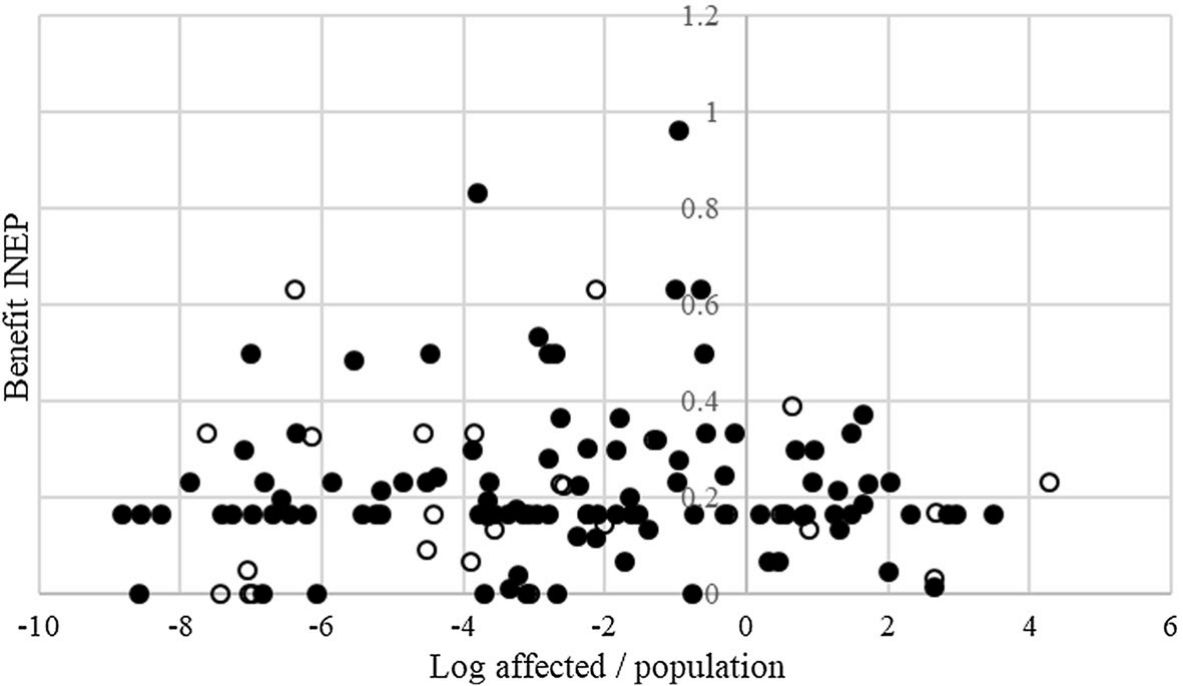
Average number of affected individuals, share of total population, versus Benefit INEP. *Notes*: See Fig. 2. The benefit INEP captures the specific benefits government enjoys once an SOE is declared

Figures 2 and 3 plot the average number of affected individuals (as a share of the total population) between 1990 and 2011, against the cost and benefit INEP, respectively. If a general feature of emergency constitutions reflects natural disaster risks, high-risk environments invariably should have more permissive provisions. We observe no such association and note, as is evident in both figures that the correlations are close to zero. As such, our data do not exhibit any clear signs of empirical relations that would induce simultaneity biases in our subsequent estimates. Our additional tests in Appendix Tables 5 and 6 also suggest that the risk environment does not affect the design of the emergency constitution, and that reverse causality is not a likely issue. We therefore interpret the events as approximately exogenous and the estimates as evidence of causal effects.

## Estimation results

Table 2 presents the main results of our analysis to determine whether the specific characteristics of an emergency constitution affect the number of people killed when a natural disaster motivates the declaration of an SOE. Subsequent tables present the results for an expanded set of dependent variables.

**Table 2:** Main effects

	1	2	3	4	5	6
Dependent variable	No. killed	No. killed	No. killed	No. killed	No. killed	No. killed
Disaster type	All	All	Bio	Geo	Hydro	Climate
Lagged dependent	0.174*** (0.024)	0.167*** (0.026)	0.135*** (0.025)	0.165*** (0.027)	0.175*** (0.031)	0.136*** (0.022)
Log affected	0.083*** (0.013)	0.079*** (0.015)	0.029*** (0.010)	0.078*** (0.015)	0.091*** (0.018)	0.035*** (0.012)
Log population	0.545*** (0.058)	0.608*** (0.065)	0.704*** (0.058)	0.614*** (0.065)	0.586*** (0.075)	0.698*** (0.058)
Log area	− 0.042 (0.038)	0.004 (0.046)	0.002 (0.040)	0.006 (0.046)	0.020 (0.054)	0.002 (0.039)
Log latitude	− 0.049 (0.080)	− 0.071 (0.084)	− 0.089 (0.073)	− 0.072 (0.084)	0.013 (0.094)	− 0.086 (0.072)
Log GDP per capita	− 0.222*** (0.065)	− 0.316*** (0.078)	− 0.197*** (0.068)	− 0.322*** (0.079)	− 0.357*** (0.088)	− 0.196*** (0.068)
Log openness	− 0.092 (0.091)	− 0.068 (0.098)	− 0.173** (0.086)	− 0.064 (0.098)	− 0.018 (0.106)	− 0.170** (0.086)
Log gov. expenditures	− 0.149 (0.106)	− 0.128 (0.113)	− 0.135 (0.099)	− 0.123 (0.113)	− 0.218* (0.126)	− 0.136 (0.099)
Capital price level	− 0.273*** (0.077)	− 0.255*** (0.079)	− 0.173*** (0.067)	− 0.254*** (0.079)	− 0.219*** (0.084)	− 0.173*** (0.067)
Federal	0.159 (0.164)	− 0.139 (0.206)	− 0.063 (0.183)	− 0.147 (0.207)	− 0.293 (0.221)	− 0.049 (0.183)
Mixed democracy	0.404** (0.194)	0.113 (0.281)	− 0.120 (0.239)	0.108 (0.281)	0.277 (0.341)	− 0.042 (0.246)
Presidential democracy	− 0.037 (0.192)	− 0.496** (0.254)	− 0.386* (0.223)	− 0.496* (0.254)	− 0.323 (0.295)	− 0.334 (0.225)
Civilian autocracy	0.183 (0.184)	− 0.198 (0.238)	− 0.205 (0.205)	− 0.199 (0.238)	− 0.064 (0.279)	− 0.141 (0.209)
Military dictatorship	0.284 (0.225)	− 0.011 (0.266)	− 0.109 (0.229)	− 0.016 (0.266)	0.159 (0.312)	− 0.045 (0.233)
Royal dictatorship	0.742** (0.356)	0.662 (0.416)	0.151 (0.354)	0.668 (0.416)	0.751 (0.534)	0.221 (0.355)
Cost INEP	− 0.115 (0.286)	− 0.395 (0.333)	− 0.215 (0.291)	− 0.372 (0.333)	− 0.315 (0.389)	− 0.279 (0.292)
Benefit INEP	0.807*** (0.296)	1.024*** (0.346)	0.619** (0.302)	1.036*** (0.346)	1.259*** (0.397)	0.652** (0.302)
Annual FE	Yes	Yes	Yes	Yes	Yes	Yes
Legal origins FE	Yes	Yes	Yes	Yes	Yes	Yes
Regional FE	Yes	Yes	Yes	Yes	Yes	Yes
Observations	1511	1254	1377	1253	1001	1372
Countries	122	99	99	99	95	100
R squared between	0.794	0.814	0.790	0.812	0.757	0.788
R squared within	0.039	0.046	0.021	0.046	0.047	0.022
Wald Chi squared	1101.10	933.04	1232.36	933.24	4640.34	1240.12

*** (**) [*] denote significance at *p* < .01 (*p* < .05) [*p* < .10]. Column 2 excludes all OECD member states

### Main results: casualties during natural disasters

Column 1 of Table 2 contains all observations, i.e., all cases found in the data where a significant natural disaster occurred, where the country had an emergency constitution, and where an emergency could be declared. It is well known that constitutional reality does not always reflect constitutional text perfectly. To avoid the possibility that results are driven by countries that supposedly are more constitution-abiding than others, all OECD member states are excluded in Column 2. Based on the full sample, Columns 3 to 6 focus on particular disasters: biological (Column 3), geophysical (Column 4), hydrological (Column 5) and climatic (Column 6).

Surmising an emergency constitution’s effectiveness in limiting the number of people killed during a natural disaster explicitly must account for the severity of the disaster. Incorporating the log of the number of people affected by a disaster seems to offer a straightforward measure of the disaster’s severity. As expected, the number of people affected is correlated highly with the number of deaths. The coefficients indicate that, on average, biological disasters are the least deadly and hydrological disasters the deadliest.

*Ceteris paribus*, the number of people killed is related directly to a country’s total population, while its land area is insignificant. Similarly, although previous studies have found latitude to be correlated highly with, e.g., income, democracy, and respect for basic rights, we find that latitude never is significant for explaining variations in the number of people killed. If latitude does have an effect, it is likely to be channeled fully through income.^15^

The fact that openness to trade is correlated only with biological and climatic disasters (which typically evolve slowly) demonstrates the importance of distinguishing between disaster types. In general, we find that the size of government is insignificant, while the relative investment price, our measure of economic distortions associated with government regulations, is associated with substantially fewer deaths.^16^ We also find that while a country’s specific organizational structure has no clear effects on the number of people killed, some forms of government do. Even when excluding OECD countries, presidential democracies are better at saving lives following natural disasters, while the apparently lesser effectiveness of royal dictatorships is driven spuriously by a few oil-rich monarchies.

When analyzing the effects of emergency constitutions, the cost INEP (how difficult it is to call a state of emergency) never reaches conventional significance levels, despite previous research showing that lower costs of calling an SOE lead to more declarations (Bjørnskov & Voigt, 2018b). Remarkably, the benefit INEP (the powers granted exceptionally to government during an SOE) are highly correlated *positively* with the number of people killed, implying that more people die the more attractive it is for government to declare an SOE. Our results indicate that the government’s special privileges (such as seizing property, censoring the press or dissolving parliament) make it less, rather than more, effective in dealing with natural disasters.^17^

Observing that the benefit INEP is correlated significantly with the number of people killed in a natural disaster, we isolate the single INEP variables to see which of the specific constitutional elements is likely to drive the result. The first three rows in Table 3 are the cost components and the last three are the benefit components. Among the benefit components, it is the possibility of suspending rights temporarily that drives the results across disaster types. Although the overall cost INEP is not significant in our previous estimations, two of the underlying variables become significant in some settings.

**Table 3:** Main effects, INEP unbundled into single variables

	1	2	3	4	5	6
Dependent variable	No. killed	No. killed	No. killed	No. killed	No. killed	No. killed
Disaster type	All	All	Bio	Geo	Hydro	Climate
*Full baseline included*						
Declaration rights	− 0.030 (0.153)	− 0.221 (0.173)	− 0.147 (0.151)	− 0.213 (0.173)	− 0.089 (0.200)	− 0.189 (0.152)
Approval rights	− 0.296 (0.188)	− 0.370* (0.201)	− 0.216 (0.175)	− 0.363* (0.202)	− 0.388* (0.233)	− 0.199 (0.175)
Conditions	0.406** (0.191)	0.426** (0.216)	0.298 (0.191)	0.434** (0.217)	0.387 (0.243)	0.278 (0.192)
Dissolution	0.221 (0.147)	0.243 (0.167)	0.066 (0.145)	0.246 (0.167)	0.452** (0.196)	0.081 (0.145)
Rights suspension	0.519*** (0.197)	0.613*** (0.227)	0.485** (0.201)	0.612*** (0.227)	0.539** (0.253)	0.450** (0.201)
Expropriation and censorship	0.223 (0.228)	0.291 (0.265)	0.250 (0.230)	0.309 (0.266)	0.298 (0.303)	0.306 (0.230)

Each column reports the main results from six different regressions. All goodness-of-fit statistics are like those reported in Table 2. Column 2 excludes OECD member states

Our results show that emergency constitutions explicitly mentioning a broad range of conditions justifying the declaration of an SOE (for geophysical disasters), as well as granting government the power to derogate basic rights, are associated with more fatalities.^18^ In estimates presented in an appendix, we interact the cost and benefit INEP indicators with a dummy capturing whether an SOE is declared to test whether it matters if the government actually announces an SOE in response to a natural disaster. None of our results indicate a significant difference in the consequences of the constitutional characteristics when an SOE has been declared or any effects of announcing an SOE per se. While none of the interactions is significant, the effects of declaring an SOE in response to a natural disaster are estimated much less precisely, which may indicate that at least some governments make effective use of SOEs, while others abuse it. As such, the purely formal procedural aspects of calling an SOE evidently are not important while some aspects of an emergency constitution affect political behavior if a de facto SOE exists.

We extend our analysis by interacting the cost and benefit INEP measures with the logarithm to the number of people affected (per 1000 inhabitants) to determine if the effects of emergency constitutions *mediate* the effects of natural disasters. While confirming previous results, the estimates indicate that, for geophysical and hydrological disasters (e.g., earthquakes, volcanic eruptions, floods and tsunamis that pose particularly immediate threats), more people are killed in countries in which the emergency constitution makes it relatively *easy* to call an SOE. When declaring an SOE is particularly easy, the estimates suggest that 15% of the affected people are killed. Conversely, although the point estimates differ, no significant association is found between the number killed and the number affected in countries hit by a climate or biological event, if the emergency constitution’s INEP cost indicators are very restrictive.

### Side effects

Because the foregoing results are counterintuitive and suggest abuses of emergency provisions by political actors during natural disasters, we explore the effects of emergency constitutions on respect for human rights, democracy and the policies adopted during disasters. Table 4 presents the results of entering an interaction between the cost INEP indicator and the logarithm of the number of people affected, allowing us to assess the conditional effects of the sizes of disasters. In a lower panel, we provide conditional estimates by entering an interaction between the logarithm of the number of people affected and the benefit INEP indicator.

**Table 4:** Side effects, policies, human rights and democracy

	1	2	3	4	5	6	7	8	9
Dependent variable	IEF	IEF RoL	IEF Gov	IEF Reg	IEF Mar	Ln conflict	CIRI Emp	CIRI phys	Xpolity
Disaster type	All	All	All	All	All	All	All	All	All
Lagged dependent	0.951*** (0.011)	0.891*** (0.012)	0.905*** (0.013)	0.865*** (0.015)	0.902*** (0.015)	0.384*** (0.025)	0.826*** (0.017)	0.680*** (0.020)	0.882*** (0.016)
Log affected	− 0.143*** (0.058)	− 0.038 (0.093)	− 0.159 (0.113)	− 0.342*** (0.107)	− 0.171 (0.112)	− 0.214*** (0.067)	0.042 (0.031)	0.017 (0.026)	− 0.008 (0.016)
Log population	0.036 (0.078)	− 0.042 (0.123)	0.051 (0.152)	0.065 (0.143)	0.167 (0.149)	0.404*** (0.087)	− 0.049 (0.041)	− 0.198*** (0.036)	0.024 (0.037)
Log area	− 0.032 (0.057)	− 0.016 (0.090)	0.116 (0.112)	− 0.022 (0.105)	− 0.114 (0.109)	0.000 (0.064)	0.002 (0.031)	0.027 (0.025)	− 0.038 (0.027)
Log latitude	0.136 (0.112)	0.497*** (0.179)	0.059 (0.219)	0.001 (0.206)	0.185 (0.216)	− 0.485*** (0.132)	0.157** (0.063)	0.151*** (0.052)	0.163*** (0.059)
Log GDP per capita	0.079 (0.109)	0.899*** (0.199)	− 0.409** (0.199)	0.164 (0.201)	0.553*** (0.201)	− 0.325*** (0.107)	0.164*** (0.053)	0.136*** (0.044)	0.089* (0.050)
Federal	− 0.209 (0.250)	− 0.051 (0.394)	0.146 (0.484)	− 0.503 (0.457)	− 1.138** (0.485)	0.993*** (0.283)	− 0.285** (0.134)	− 0.365*** (0.111)	− 0.176 (0.113)
Mixed democracy	− 0.028 (0.308)	− 0.491 (0.485)	− 0.307 (0.598)	− 0.759 (0.561)	0.213 (0.589)	− 0.305 (0.349)	− 0.089 (0.167)	0.094 (0.136)	0.106 (0.134)
Presidential democracy	0.070 (0.283)	− 0.314 (0.452)	− 0.515 (0.551)	− 0.601 (0.531)	0.572 (0.544)	− 0.414 (0.321)	− 0.136 (0.155)	0.027 (0.126)	0.122 (0.139)
Civilian autocracy	0.023 (0.286)	− 1.100** (0.456)	− 0.681 (0.532)	− 0.989* (0.512)	0.046 (0.541)	− 0.074 (0.306)	− 0.601*** (0.163)	− 0.232* (0.123)	− 0.413** (0.163)
Military dictatorship	0.248 (0.348)	− 1.029* (0.559)	0.631 (0.655)	− 1.251** (0.629)	0.139 (0.666)	− 0.392 (0.374)	− 0.447** (0.199)	− 0.216 (0.148)	− 0.345 (0.202)
Royal dictatorship	0.187 (0.549)	− 0.162 (0.869)	− 0.961 (1.074)	0.753 (1.011)	1.329 (1.055)	− 0.436 (0.632)	− 0.308 (0.312)	0.012 (0.250)	− 0.431 (0.308)
Cost INEP	− 2.284* (1.250)	− 0.971 (1.993)	− 2.319 (2.433)	− 5.558** (2.295)	− 2.940 (2.411)	− 3.178** (1.477)	0.799 (0.699)	− 0.314 (0.575)	− 0.089 (0.199)
Benefit INEP	− 0.041 (0.429)	0.448 (0.682)	− 0.851 (0.835)	− 0.184 (0.791)	− 0.595 (0.823)	1.619*** (0.497)	0.007 (0.233)	− 0.261 (0.192)	0.068 (0.203)
Affected * Cost INEP	0.232** (0.115)	0.112 (0.184)	0.324 (0.223)	0.522** (0.211)	0.284 (0.221)	0.443*** (0.135)	− 0.084 (0.064)	− 0.035 (0.052)	0.082** (0.040)
Annual FE	Yes	Yes	Yes	Yes	Yes	Yes	Yes	Yes	Yes
Legal origins FE	Yes	Yes	Yes	Yes	Yes	Yes	Yes	Yes	Yes
Regional FE	Yes	Yes	Yes	Yes	Yes	Yes	Yes	Yes	Yes
Observations	1033	1033	1033	1033	1033	1354	1288	1288	1349
Countries	109	109	109	109	109	119	118	118	110
R squared between	0.676	0.609	0.488	0.637	0.564	0.074	0.402	0.226	0.585
R squared within	0.992	0.996	0.983	0.972	0.989	0.796	0.983	0.955	0.985
Wald Chi squared	19,578.71	31,551.80	10,737.67	7748.82	9990.41	751.63	9966.64	3755.20	15,742.23
*Conditional estimates*									
Benefit INEP	2.992** (1.349)	2.276 (2.140)	4.269 (2.633)	5.049** (2.489)	1.106 (2.601)	0.185 (1.589)	− 0.147 (0.743)	− 0.138 (0.611)	0.001 (0.208)
Affected * Benefit INEP	− 0.311** (0.131)	− 0.187 (0.208)	− 0.526** (0.256)	− 0.538** (0.243)	− 0.175 (0.253)	0.143 (0.153)	0.016 (0.071)	− 0.012 (0.058)	− 0.037 (0.044)

*** (**) [*] denote significance at *p* < .01 (*p* < .05) [*p* < .10]

Focusing on the effects of natural disasters on economic policies and institutions, we rely on the Heritage Foundation’s Index of Economic Freedom (IEF), whose values range from 0 to 100, with larger values indicating more economic freedom. In Column 1, we report results drawing on the entire Index; subsequent columns report estimates for the subindices rule of law (RoL), government size (Gov), regulatory activity (Reg) and market openness (Mar). The control variables exhibit substantial persistence over time, consistent with previous evidence (cf. Sobel & Coyne, 2011). Also consistent with previous studies, we find that the rule of law is associated significantly with latitude, that richer countries tend to develop larger government sectors (the negative estimate in Column 3) and more openness towards international markets (Column 5), and that autocracies tend to be more regulated.

The results in the first columns of Table 4 show that as the magnitude of the disaster increases and when the cost INEP measure is large, more protection is granted to citizens. The opposite is the case for the benefit INEP measure for both regulatory activity and government size, suggesting that the more benefits granted to the executive branch by the emergency constitution, the more regulatory activity and spending increase.

Focusing on conflicts and human rights, we again observe substantial persistence over time. Conflicts are more frequent in poorer countries closer to the equator, while we observe substantial evidence for the opposite pattern for both types of human rights and democracy. We also see more conflicts and less respect for physical integrity rights in more populous countries, as well as in federal states and civilian autocracies. Consistent with previous studies, we find less repression in countries further from the equator. We also find that countries with larger government sectors suspend or suppress empowerment rights substantially more often.

Most pertinently, we find that the number of conflicts created by disasters is increasing in the benefit INEP measure.^19^ While the ease with which an SOE can be declared is associated negatively with conflict, the opposite association with the benefit INEP may shed light on the finding that more people are killed when the emergency constitution allows basic rights to be undermined. The conditional effects of the sizes of disasters given the benefit INEP in the bottom panel show that physical integrity rights are repressed more substantially in more serious disasters in countries with SOEs that offer more benefits to the executive. We consider that result to confirm our counterintuitive finding that political actors in certain countries abuse emergency provisions during natural disasters.

All of our findings are unaffected by whether or not a country actually declares an SOE in response to a natural disaster, indicating that emergency constitutions do not offer positive effects that contribute to saving lives in natural disasters. We do, however, find evidence that a government’s reaction is affected by the emergency provisions to which they are subject *de jure*. The existence of an emergency constitution seems to have significant consequences, although perhaps not the officially intended consequences. Our evidence on the side effects of emergency constitutions indicates that rather than enabling governments to deal effectively with disasters, and in particular limiting the number of fatalities, most governments use them for other purposes. What those purposes are, and more generally how to interpret our findings, is the topic of our final section.

## Conclusions and open questions

Emergency constitutions have been adopted since ancient Roman times with the normative justification that it is necessary to allow the executive branch to bypass the separation of powers and supply it with additional powers in order to counter the consequences of emergencies. While some emergencies are a consequence of government action, natural disasters (floods, earthquakes, epidemics and extreme weather conditions) are random acts of nature, making them exogenous and the settings for natural experiments testing whether emergency constitutions deliver on their implicit promises.

We ask whether the characteristics of an emergency constitution affect its effectiveness in minimizing the casualties caused by a natural disaster and explore its side effects. Relying on a panel of up to 1511 observations from 122 countries affected by at least one natural disaster touching some of the population, we estimate how many people are killed in natural disasters relative to how many are affected, and how two separable components of emergency constitutions influence the death toll. We rely on the same set of natural disasters to estimate the effects of a given emergency constitution on respect for human rights, democracy and conflict intensity, relying on a set of indicators for economic policy and political institutions.

We find that when emergency constitutions allocate more powers to the executive branch, natural disasters kill more people. That empirical finding suggests clearly that granting additional powers to the chief executive not only is ineffective but also can have unintended consequences. Our finding that the ease with which an SOE can be declared results in more intense civil conflicts following more serious disasters lends credibility to that interpretation. We also observe that when the emergency constitution allocates more power to the executive branch, governments exploit natural disasters as pretexts for increasing market regulations and spending. Allocating more discretionary power to the chief executive thus may undermine private disaster relief efforts (cf. Skarbek, 2014).

Given our analysis, one might ask why emergency constitutions that do not fulfill their intended purposes, and have a deplorable impact on human rights, are not redesigned or abandoned. A simple answer is that emergency constitutions are attractive politically. For example, the power to censor media may be a way to remain in power, despite obvious negative consequences (Leeson, 2008). In a few cases (e.g., Austria following WW II), those shortcomings are sufficient to compel the constitutional assembly to terminate the emergency constitution. Our analysis suggests a concrete way of redesigning emergency constitutions. Specifically, limit the power of a government that has declared an SOE to suspend property and basic human right, even if that constraint is inconsistent with the incentives of most political actors. In other words, an accurate description of the reality of emergency constitutions is inconsistent with much standard legal work in the area and more likely to be explained by public choice theory.

Given the questionable performance of emergency constitutions with respect to dealing with the immediate consequences of natural disasters as well as deplorable human rights records, Voigt (2021) asks whether their adoption can even be justified in the first place. Taking a social-contract perspective, he suggests that emergency provisions could protect politicians and citizens from the urge “to do something” after a natural disaster (or terrorist event), saving them from action bias and succumbing to time-inconsistent preferences. In principle, emergency constitutions also make government behavior more predictable. Using statutory law to enact emergency provisions tends to make those measures permanent, whereas emergency constitutions provide some assurance that the provisions are temporary.

Once a decision in favor of an emergency constitution has been made, its main traits need to be chosen. Given the results reported herein, limiting the benefits allocated to governments under SOEs seems warranted. That recommendation refers especially to the possibility of suspending rights, which ideally should be kept to a minimum. However, we also must note that such constitutional restraint likely is inconsistent with the incentives of most political actors.

The present paper takes only a first step toward assessing the effectiveness of emergency constitutions. The content of emergency constitutions may affect the survival of a country’s constitutional order in total. Lührmann and Rooney (2020) show that an SOE declaration frequently is the starting point of democratic decline and interpret their findings in a way fully consistent with ours. We already have observed that natural disasters can be pretexts for politicians to pursue more easily their own agendas because of looser constraints on action. But not all SOEs incentivize democratic decline; another issue that deserves attention is the number of years it takes to return to the status quo ante, if that is possible.

## Appendix for “emergencies: on the misuse of government powers”

See Tables 5, 6 and 7.

**Table 5:** Predicting emergency provisions

	INEP		Cost INEP		Benefit INEP	
Log latitude	− 0.026 (0.017)	0.013 (0.021)	− 0.067*** (0.019)	− 0.016 (0.027)	0.014 (0.023)	0.042 (0.030)
Log area	− 0.006 (0.007)	− 0.008 (0.007)	− 0.007 (0.009)	− 0.005 (0.010)	− 0.005 (0.009)	− 0.011 (0.010)
Landlocked	− 0.050 (0.039)	− 0.048 (0.041)	− 0.125** (0.050)	− 0.120** (0.052)	0.025 (0.057)	0.025 (0.061)
High elevation	0.000** (0.000)	0.000** (0.000)	0.000 (0.000)	0.000 (0.000)	0.000 (0.000)	0.000 (0.000)
Low elevation	− 0.000* (0.000)	− 0.000* (0.000)	− 0.000 (0.000)	− 0.000 (0.000)	− 0.000 (0.000)	0.000 (0.000)
Volcano zone	− 0.006 (0.029)	− 0.017 (0.033)	− 0.039 (0.036)	− 0.053 (0.044)	0.029 (0.046)	0.019 (0.044)
Coastline	0.010 (0.031)	0.006 (0.058)	0.029 (0.045)	0.016 (0.089)	− 0.008 (0.045)	− 0.005 (0.063)
Democracy 1950–60	− 0.005* (0.003)	− 0.003 (0.005)	− 0.009** (0.004)	− 0.005 (0.007)	− 0.001 (0.004)	− 0.000 (0.005)
Coups 1950–60	0.011 (0.012)	0.008 (0.011)	0.016 (0.018)	0.004 (0.020)	0.006 (0.017)	0.012 (0.019)
Average disaster affected 1960–1980	0.000 (0.001)	0.000 (0.001)	0.001 (0.001)	0.000 (0.001)	0.000 (0.001)	0.000 (0.001)
Legal origins FE	No	Yes	No	Yes	No	Yes
Region FE	No	Yes	No	Yes	No	Yes
Observations	115	115	115	115	115	115
R squared	0.122	0.208	0.219	0.291	0.048	0.110
F statistic	2.92	4.89	6.54	7.18	0.79	1.92
Root MSE	0.136	0.135	0.184	0.184	0.170	0.172

*** (**) [*] denote significance at *p* < .01 (*p* < .05) [*p* < .10]. Legal origins FE include dummies for civil, common and Scandinavian law; and region FE include dummies for Asia, Latin America and the Caribbean, the Middle East and North Africa, and Sub-Saharan Africa

**Table 6:** Main effects, dependent on calling an SOE

	1	2	3	4	5	6
Dependent variable	No. killed	No. killed	No. killed	No. killed	No. killed	No. killed
Disaster type	All	All	Bio	Geo	Hydro	Climate
Lagged dependent	0.175*** (0.024)	0.167*** (0.026)	0.134*** (0.025)	0.165*** (0.026)	0.175*** (0.031)	0.134*** (0.025)
	*Full baseline included*					
Cost INEP	− 0.170 (0.310)	− 0.509 (0.368)	− 0.379 (0.319)	− 0.483 (0.368)	− 0.452 (0.439)	− 0.476 (0.320)
Benefit INEP	0.792** (0.309)	1.089*** (0.364)	0.626** (0.318)	1.099*** (0.364)	1.242*** (0.419)	0.663** (0.318)
SOE called	− 0.024 (0.368)	− 0.068 (0.437)	− 0.274 (0.390)	− 0.054 (0.437)	− 0.367 (0.488)	− 0.383 (0.389)
SOE * Cost INEP	0.238 (0.689)	0.517 (0.795)	0.828 (0.712)	0.494 (0.796)	0.632 (0.883)	1.043 (0.711)
SOE * Benefit INEP	0.055 (0.782)	− 0.564 (0.895)	− 0.127 (0.798)	− 0.556 (0.895)	0.242 (0.977)	− 0.111 (0.796)
Observations	1511	1254	1377	1253	1001	1372
Countries	122	99	99	99	95	100
R squared between	0.794	0.814	0.790	0.812	0.759	0.788
R squared within	0.039	0.047	0.021	0.047	0.048	0.024
Wald Chi squared	1100.12	932.37	1233.35	932.54	4629.28	1243.11
*Effects at SOE called*						
Cost INEP	0.067 (0.639)	0.008 (0.723)	0.448 (0.651)	0.011 (0.723)	0.180 (0.787)	0.567 (0.649)
Benefit INEP	0.847 (0.751)	0.525 (0.857)	0.499 (0.763)	0.542 (0.858)	0.1484 (0.936)	0.552 (0.761)

*** (**) [*] denote significance at *p* < .01 (*p* < .05) [*p* < .10]

**Table 7:** Main effects of size of disaster

	1	2	3	4	5	6	7	8	9	10
Dependent variable	No. Killed	No. killed	No. killed	No. killed	No. killed	No. killed	No. killed	No. killed	No. killed	No. killed
Disaster type	All	All	Bio	Bio	Geo	Geo	Hydro	Hydro	Climate	Climate
Lagged dependent	0.168*** (0.026)	0.166*** (0.026)	0.135*** (0.025)	0.134*** (0.025)	0.166*** (0.026)	0.164*** (0.026)	0.176*** (0.031)	0.173*** (0.031)	0.134*** (0.025)	0.133*** (0.025)
*Full baseline included*										
Cost INEP	− 0.497 (0.339)	− 0.400 (0.333)	− 0.202 (0.291)	− 0.223 (0.291)	− 0.475 (0.339)	− 0.378 (0.334)	− 0.652 (0.409)	− 0.344 (0.391)	− 0.258 (0.292)	− 0.278 (0.292)
Benefit INEP	1.032*** (0.346)	1.017*** (0.346)	0.623** (0.302)	0.672** (0.305)	1.046*** (0.346)	1.028*** (0.346)	1.262***(0.396)	1.238*** (0.397)	0.662** (0.302)	0.707** (0.305)
Log no. affected per 1000	0.027 (0.036)	0.071*** (0.024)	0.001 (0.026)	0.012 (0.017)	0.024 (0.036)	0.068*** (0.024)	− 0.019 (0.046)	0.066** (0.027)	− 0.001 (0.026)	0.014 (0.017)
Affected * Cost INEP	0.119 (0.073)		0.062 (0.053)		0.122* (0.074)		0.248*** (0.095)		0.069 (0.053)	
Affected * Benefit INEP		0.039 (0.086)		0.079 (0.062)		0.043 (0.087)		0.113 (0.097)		0.077 (0.062)
Observations	1254	1254	1377	1377	1253	1253	1001	1001	1372	1372
Countries	99	99	99	99	99	99	95	95	100	100
R squared between	0.815	0.815	0.789	0.792	0.814	0.813	0.757	0.759	0.786	0.789
R squared within	0.048	0.046	0.023	0.022	0.048	0.046	0.055	0.049	0.024	0.022
Wald Chi squared	936.99	932.63	1234.13	1234.55	937.30	932.89	-		1243.05	1242.71
*Effects of size evaluated at*										
10th decile	0.047* (0.025)	0.071***								
(0.024)	0.011 (0.018)	0.012 (0.017)	0.044* (0.025)	0.068*** (0.024)	0.022 (0.032)	0.066** (0.027)	0.011 (0.018)	0.014 (0.017)		
25th decile	0.071*** (0.016)	0.078*** (0.016)	0.024** (0.011)	0.026** (0.011)	0.069*** (0.016)	0.076*** (0.016)	0.072*** (0.019)	0.085*** (0.018)	0.025** (0.011)	0.027** (0.011)
Median	0.081*** (0.015)	0.078*** (0.016)	0.029*** (0.010)	0.026** (0.011)	0.079*** (0.015)	0.076*** (0.016)	0.093*** (0.017)	0.085*** (0.018)	0.030*** (0.010)	0.027** (0.011)
75th decile	0.099*** (0.019)	0.080*** (0.015)	0.038*** (0.013)	0.031*** (0.010)	0.097*** (0.019)	0.078*** (0.015)	0.129*** (0.023)	0.093*** (0.018)	0.040*** (0.013)	0.032*** (0.010)
90th decile	0.110*** (0.024)	0.091*** (0.029)	0.044*** (0.016)	0.052** (0.020)	0.109*** (0.024)	0.089*** (0.029)	0.153*** (0.029)	0.122*** (0.033)	0.047*** (0.016)	0.052*** (0.021)

*** (**) [*] denote significance at *p* < .01 (*p* < .05) [*p* < .10]

### Notes on reverse causality

A potential problem in this paper is that the design of provisions in the emergency constitution might reflect the threat level of a country. This would happen if, for example, large or particularly destructive natural disasters lead politicians to implement provisions that make it easy to call a state of emergency, or provide the executive with specific powers during emergencies. In that case, our empirical results would suffer from endogeneity bias.

We have no way of instrumenting aspects of the emergency constitution captured in the two INEP indicators, as there is very little literature on what determines the design of an emergency constitution, and we have been unable to find any variables that are sufficiently strong to work as valid instruments. As a viable alternative, we follow Bjørnskov and Voigt (2020) and test directly for the reverse causality direction. Table 8 shows the results of two types of tests. In Columns 1, 2, 5 and 6, we predict the cost and benefit INEP with three lags of the log to the number of disasters and the number of people killed during those disasters. In Columns 3, 4, 7 and 8 we use the average number of disasters and deaths in the 1990s to predict the observed 2011 cost and benefit INEP. In the first set of tests, the sample includes 58 observations where the emergency constitution changed during our period of observation. In the second set of tests, the sample is a simple cross-section of all 118 countries with full data from the 1990s. In all cases, we control for the log to population size.

**Table 8:** Reverse causality tests explaining emergency constitutions

	Cost INEP				Benefit INEP			
	1	2	3	4	5	6	7	8
Log distasters, -1	0.019 (0.042)				0.088* (0.049)			
Log distasters, -2	0.002 (0.041)				0.033 (0.047)			
Log distasters, -3	0.025 (0.039)				− 0.036 (0.039)			
Log disasters, average			− 0.029 (0.042)				− 0.032 (0.029)	
Log killed, -1		0.016 (0.010)				0.012 (0.010)		
Log killed, -2		− 0.011 (0.009)				− 0.003 (0.011)		
Log killed, -3		0.014* (0.007)				0.011 (0.008)		
Log average killed				0.025** (0.011)				0.011 (0.010)
Log population	0.009 (0.015)	− 0.017 (0.017)	− 0.002 (0.014)	− 0.032** (0.014)	− 0.036** (0.014)	− 0.026 (0.017)	0.011 (0.011)	− 0.006 (0.012)
Observations	58	58	118	118	58	58	118	118
Within R squared	0.006	0.226	0.010	0.005	0.260	0.053	0.006	0.001
F statistic	1.58	7.52	0.61	3.00	8.20	3.11	0.68	0.60

As the estimates suggest, we find no consistent evidence that the INEP measures are affected by the threat level. Of the 12 lag estimates, two are weakly significant, but the estimates frequently change signs. The log to the average number of people killed in natural disasters in the 1990s attains significance at the 5% level when the Cost INEP is the dependent variable. However, further tests suggest that this finding is driven by only few observations, and the estimate per se is very small. As our main findings relate to the benefit INEP, we tentatively assess that these results do not indicate any major problems of reverse causality.

Given that causality can be established, we provide another set of robustness tests in Table 9. Column 1 presents our estimates using the full sample for comparison. In the subsequent columns, we provide estimates for samples including only autocracies (Column 2), only democracies (Column 3), only countries in Latin American and the Caribbean (Column 4), only countries in Sub-Saharan Africa (Column 5), and only countries without a communist past (Column 6). We find that main control variables are relatively stable across the five robustness tests. The exceptions are the lagged dependent, which is far from significance in the very small Latin American sample, and GDP per capita, which is highly significant in the autocratic sample, but falls to insignificance in the Latin American sample, and is both very small and insignificant in the Sub-Saharan African sample.

**Table 9:** Main effects, all countries versus specific groups

	1	2	3	4	5	6
Dependent variable	No. killed	No. killed	No. killed	No. killed	No. killed	No. killed
Sample	All	Autocracies	Democracies	Latin America	Sub-Saharan Africa	No post-communist
Lagged dependent	0.174*** (0.024)	0.205*** (0.036)	0.117*** (0.033)	− 0.003 (0.059)	0.193*** (0.041)	0.172*** (0.025)
Log affected	0.083*** (0.013)	0.056*** (0.022)	0.115*** (0.017)	0.185*** (0.032)	0.065** (0.026)	0.106*** (0.015)
Log population	0.545*** (0.058)	0.530*** (0.089)	0.552*** (0.084)	0.482** (0.246)	0.576*** (0.114)	0.522*** (0.062)
Log area	− 0.042 (0.038)	0.053 (0.069)	− 0.083 (0.050)	− 0.132 (0.162)	0.009 (0.078)	− 0.069* (0.040)
Log latitude	− 0.049 (0.080)	− 0.206* (0.115)	0.145 (0.125)	0.137 (0.238)	− 0.206* (0.109)	− 0.053 (0.080)
Log GDP per capita	− 0.222*** (0.065)	− 0.296*** (0.098)	− 0.183* (0.101)	− 0.521 (0.433)	− 0.024 (0.125)	− 0.258*** (0.069)
Log openness	− 0.092 (0.091)	0.004 (0.114)	− 0.252 (0.179)	− 0.293 (0.413)	− 0.166 (0.128)	− 0.094 (0.093)
Log gov. expenditures	− 0.149 (0.106)	− 0.282* (0.167)	− 0.056 (0.153)	− 0.484 (0.507)	− 0.270 (0.184)	− 0.204* (0.112)
Capital price level	− 0.273*** (0.077)	− 0.159* (0.090)	− 0.342 (0.249)	− 0.321 (0.632)	− 0.041 (0.118)	− 0.336*** (0.087)
Federal	0.159 (0.164)	− 0.165 (0.296)	0.258 (0.201)	− 0.695 (0.690)	− 0.377 (0.337)	0.127 (0.171)
Mixed democracy	0.404** (0.194)	–	0.348* (0.208)	–	− 1.121 (1.854)	0.353 (0.222)
Presidential democracy	− 0.037 (0.192)	–	0.046 (0.222)	0.053 (1.172)	− 1.438 (1.796)	− 0.048 (0.199)
Civilian autocracy	0.183 (0.184)	− 0.879 (0.904)	–	0.857 (1.138)	− 1.356 (1.822)	0.209 (0.201)
Military dictatorship	0.284 (0.225)	− 0.831 (0.911)	–	–	− 1.017 (1.804)	0.290 (0.234)
Royal dictatorship	0.742** (0.356)	–	–	–	− 2.096 (2.007)	0.746** (0.359)
Cost INEP	− 0.115 (0.286)	− 0.724 (0.552)	0.343 (0.366)	0.573 (0.991)	− 1.444* (0.783)	− 0.112 (0.316)
Benefit INEP	0.807*** (0.296)	1.907*** (0.460)	− 0.251 (0.418)	− 1.331 (1.771)	1.167** (0.483)	0.933*** (0.322)
Annual FE	Yes	Yes	Yes	Yes	Yes	Yes
Legal origins FE	Yes	Yes	Yes	Yes	Yes	Yes
Regional FE	Yes	Yes	Yes	Yes	Yes	Yes
Observations	1511	684	827	256	526	1321
Countries	122	71	73	20	39	102
R squared between	0.794	0.753	0.776	0.922	0.786	0.807
R squared within	0.039	0.062	0.065	0.289	0.133	0.059
Wald Chi squared	1101.10	–	–	–	–	–

*** (**) [*] denote significance at *p* < .01 (*p* < .05) [*p* < .10]. Column 2 excludes all OECD member states

Nevertheless, the main outcome of these tests is that the effects of an emergency constitution are specific to autocracies. While the estimated effect of the Benefit INEP is relatively small and insignificant for democracies, it is rather large and precisely measured for autocracies. This difference is consistent with the insignificant Latin American estimate, as countries in the region with full data since 1990 were almost exclusively democratic. It is also consistent with the slightly larger estimate for Sub-Saharan Africa, in which approximately only 15% of the countries within the period were democratic.

## Footnotes

1 The examples are described in more detail in Bjørnskov and Voigt (2021).

2 Leeson and Sobel (2008) show that natural disasters in the United States trigger federal natural disaster relief creating resource windfalls for the affected states. Such federal relief strengthens incentives for fraudulent appropriation at the state level. Leeson and Sobel also find that disaster-prone states receive more disaster relief and, in turn, become more corrupt. The ratchet effect describes a scenario wherein government involvement provoked by a crisis does not return public spending to the pre-crisis level. Robert Higgs (1987) and analyzes such a “ratchet” effect in detail for the United States.

3 The greater predictability of government behavior under a state of emergency might be an additional consideration.

4 Because terrorist incidents can be viewed as reactions to government policies, voters might interpret the declaration of an SOE as an indication that government policies have been unsuccessful. Bjørnskov and Voigt (2020) show that declaring an SOE after a terrorist attack is significantly less likely in election years, supposedly because politicians believe that it would reduce their popularity. Conversely, natural disasters are exogenous events even though their severity may not be perfectly so. Floods and earthquakes are, for example, less likely to be severe events where governments or private initiatives have built dikes and reinforced buildings. However, we show that the design of emergency constitutions generally is not associated with the likelihood of a specific event.

5 Biological disasters include epidemics such as malaria or Ebola, geophysical ones (sometimes also called geological) include earthquakes and volcanic activity, hydrological ones include floods and landslides and climatological events include droughts and wildfires.

6 International law constrains such suspensions and some core basic rights cannot be limited even during a state of emergency. According to article 4(2) of the ICCPR, these are the right to life, to freedom from torture, to be free from slavery, to be free from the retroactive application of penal laws, and the right to freedom of thought, conscience and religion.

7 The determinants of SOE declarations are studied in Bjørnskov and Voigt (2018b).

8 It is worth noting that the necessity of reaching consensus gives opposition parties opportunities to hijack the disaster by forcing acceptance of their policies in exchange for consent to emergency measures.

9 Our results are quite robust, even when different thresholds are adopted.

10 The 16 categories are: occurrence of a coup, state of emergency or martial law, political or extrajudicial killing, disappearances, torture, people imprisoned owing to their political, religious or other beliefs, judicial independence, government censorship of the media, official state religion, restrictions on religious practices, domestic and foreign travel, political participation, union activities, women’s political rights, women’s economic rights, and social equality of women.

11 The construction of the index, the development of index values over time, and typical combinations of the six variables are discussed in detail in Bjørnskov and Voigt (2018a).

12 In other contexts, such a fine-grained delineation has proved important. When comparing presidential and parliamentary democracies, presidential systems seem to generate less government revenue, have lower central government expenditure and suffer from more corruption [Appendix 2 in Voigt (2020) summarizes these findings]. The distinction also might be relevant in our context because presidents cannot be forced out of office by a no-confidence vote and therefore might act more vigorously than prime ministers.

13 While we are aware that entering a lagged dependent variable gives rise to Nickell bias, we believe it is relatively unimportant here. As the size of the bias is approximately 1/T, where T is the length of the panel, the actual bias is about 5%, which does not warrant raising the other problems associated with estimators that can alleviate the Nickell bias.

14 In previous research, we find that the specific characteristics of the emergency constitution are not affected by the frequency of natural disasters (Bjørnskov & Voigt, 2017). The same conclusion holds for the probability of exercising an emergency constitution per se. We provide empirical evidence in an appendix suggesting strongly that emergency provisions are not affected by the threat environment. Specifically, we capture the severity of the threat environment by accounting for the average number of citizens affected by natural disasters in the period between 1960 and 1980, if the country is in a volcano zone, has a long coastline and its elevation is particularly low or mountainous. We subsequently show that the same factors are not associated significantly with any of the INEP measures.

15 Latitude also can be a proxy for institutional quality or state capacity when more direct measures are unavailable. We do not rely on direct measures because they would be ‘bad controls’ when the emergency constitution either affects or reflects state capacity or overall institutional quality. However, in an additional set of tests (not reported but available upon request) we enter economic freedom into the specification, treating rule of law as a direct measure of institutional quality and government size as a proxy for state capacity. We find no robust evidence for direct effects of institutions as measured by the Heritage Foundation (2016).

16 Our interpretation of the relative price of capital rests on the idea that countries with low investment costs also have in place government regulations and price controls (including legislation against price gouging) that limit the supply of the capital equipment necessary to deal with emergencies and thereby constrain the ability of the private sector to respond to disasters.

17 To alleviate concerns that the results might be driven by Latin American countries, we run a robustness test distinguishing between democracies and autocracies, including separate estimates for both Sub-Saharan Africa and Latin America. It turns out that our findings actually are driven by autocracies. The results are reported in more detail in the “Appendix Table 9”.

18 Although we observe indications that approval rights are associated negatively with the death toll, an association that is significant at the 5% level for geophysical and hydrological disasters (when OECD member countries are excluded), we must warn that applying a Bonferoni correction for multiple test bias leaves the estimates only weakly significant.

19 The results also indicate that democratization is more likely during larger disasters when the cost INEP is higher. We have, however, been unable to replicate that result with other indicators of democracy, such as the original Polity IV index, or the dichotomous DD indicator from Cheibub et al. (2010).
